# The ubiquitin ligase CHIP modulates cellular behaviors of gastric cancer cells by regulating TRAF2

**DOI:** 10.1186/s12935-019-0832-z

**Published:** 2019-05-16

**Authors:** Hanjue Dai, Hao Chen, Jingjing Xu, Jun Zhou, Zhili Shan, Hengying Yang, Xiaojun Zhou, Feng Guo

**Affiliations:** 1 Oncology center, Changzhou Second People’s Hospital Affiliated Nanjing Medical University, Changzhou, 213003 China; 2 Department of Oncology, The Second People’s Hospital of Taizhou, Taizhou, 225500 China; 3 Center for Clinical Laboratory, The First Affiliated Hospital of Soochow University, Suzhou, 215006 China; 4 Department of General Surgery, The First Affiliated Hospital of Soochow University, Suzhou, 215006 China; 5 Department of Oncology, Nanjing Medical University Affiliated Suzhou Hospital, Baita West Road 16, Suzhou, 215001 China

**Keywords:** Gastric cancer, CHIP, TRAF2, Cellular behaviors, Prognostic factor

## Abstract

**Background:**

CHIP is an E3 ubiquitin ligase that plays contrast roles in diverse human malignancies, depending on its targets. To date, the mechanisms underlying the function of CHIP in gastric cancer remains unclear. Here, we aim to further clarify the effects of CHIP on the development and progression of gastric cancer and explore its potential target.

**Methods:**

Stably transfected CHIP-shRNA and TRAF2-shRNA AGS gastric cancer cell lines were established using Lipofectamine 2000. Cell growth was measured by an xCelligence real-time monitoring system and colony formation assay. Cell proliferation was detected using CCK-8, Ki-67, or CFSE assays. Apoptosis was detected by TUNEL assay or Annexin V/PI-staining followed by flow cytometric analysis. Cell cycle distribution was detected by PI-staining followed by flow cytometric analysis. Cell migration and invasion abilities were measured by a real-time xCelligence system, Transwell insert, and scratch assays. The expression of cell cycle-related proteins, apoptosis-related proteins, AKT, ERK, NF-κB signaling subunits, MMP2, MMP9, and Integrin β-1 were detected by Western blotting analysis. NF-κB DNA-binding capability was quantified using an ELISA-based NF-κB activity assay. Gastric cancer tissue microarray was analyzed to investigate the expression of both CHIP and TRAF2, and their clinical significance.

**Results:**

The CHIP-silencing in the AGS cells was oncogenic evidenced by the appearance of capable of anchorage-independent growth. The CHIP-silencing significantly enhanced the AGS cell proliferation capability likely due to the induced phosphorylation of ERK. The CHIP-silencing significantly inhibited apoptosis due to increased expression of Bcl-2. The CHIP-silencing promoted the AGS cell migration and invasion abilities, likely by regulating the expression of Integrin β-1. TRAF2 expression was markedly decreased in the CHIP-overexpressing cells at protein level, but not at mRNA level. The TRAF2-silencing markedly inhibited the proliferation ability of the AGS cells, the defected cell proliferation and enhanced apoptosis were involved in. The TRAF2-silencing also attenuated the cell migration and invasion capacities of the AGS cells. Furthermore, the expression of CHIP was downregulated while the expression of TRAF2 was upregulated in gastric cancer tissues. TRAF2 expression is independent prognostic factors of gastric cancer. The expression of CHIP and TRAF2 was negatively correlated in the gastric cancer tissue. Lower CHIP or higher TRAF2 was significantly linked to shorter overall survival in gastric cancer patients.

**Conclusions:**

TRAF2 influenced diverse aspects of cellular behavior of gastric cancer cells, including cell growth, migration, and invasion, which was in contrast to the functions of CHIP. TRAF2 could be considered as an independent prognostic factor in gastric cancer patients. It is possible that TRAF2 was a substrate of CHIP and CHIP regulated the TRAF2/NF-κB axis, which modulated diverse cellular behaviors in the AGS gastric cancer cells.

**Electronic supplementary material:**

The online version of this article (10.1186/s12935-019-0832-z) contains supplementary material, which is available to authorized users.

## Background

Gastric cancer (GC) is a biologically heterogeneous disease that evolves in the background of diverse genetic and epigenetic alterations. Despite of a global reduction in the incidence of GC, the incidence (31.8 new cases per 100,000 people) in China remains high. GC ranks the fourth common malignant tumor in man and the fifth in woman. GC is the third leading cause of cancer-associated death in China [1]. Due to the asymptomatic early phase of GC, most of the patients are diagnosed at the advanced stage. The surgical resection of localized GC with the removal of adjacent lymph nodes is the only curative strategy, and patients with pathological stage II or III requires postoperative adjuvant chemotherapy [2]. Given the limited benefits of conventional chemotherapy for metastatic GC, the additions of human epidermal growth factor receptor-2 (HER-2)-targeted therapy, anti-vascular endothelial growth factor receptor 2 (VEGFR2)-targeted antibody, and immune checkpoint inhibitors are under investigations [3]. However, advances in the overall survival (OS) of patients suffering from GC are much slower than patients with other cancers. A better understanding of the molecular characteristic of metastatic GC is in need.

Carboxyl terminus of Hsc70-interacting protein (CHIP), also known as STIP1 homology and U-box containing protein 1 (STUB1), is a 34.5 kDa cytosolic protein. CHIP is highly conserved across species and is expressed preferentially in adult striated muscle and brain [4]. It is composed of amino-terminal region contains three tandem tetratricopeptide repeat (TPR) domains, a U-box containing E3 ligase, and a central part. TPR is essential for appropriate regulation of protein folding and transport. TPR-containing proteins participate in interaction with major members of the heat shock protein family, such as chaperones heat shock protein 70 (HSP70) and chaperones heat shock protein (HSP90). CHIP mediates the ubiquitination and degradation of chaperone-bound and misfolded proteins [4]. According to previous studies, CHIP functions controversially in diverse human malignancies. CHIP could function as a tumor suppressor by regulating and degrading epidermal growth factor receptor (EGFR) in pancreatic cancer [5], RelA in gastrointestinal cancer [6, 7], and c-Myc in glioma [8]. In contrast, CHIP is involved in the carcinogenesis of prostate, colon, thymus, and esophagus [9–12]. Collectively, CHIP can function as an oncogene or a tumor suppressor, depending on its specific targets.

Very few studies have reported the role of CHIP in GC. The expression of CHIP is relatively low in GC tissues. CHIP is implicated in suppressing the proliferative and metastatic potential of gastric cancer cells and is negatively correlated with tumor progression of gastric cancer [13, 14]. However, the mechanisms underlying the regulation of CHIP in GC cells remain unknown.

In this study, we investigated the biological roles of CHIP in AGS gastric cancer cells. The CHIP-silencing promoted cell growth, migration and invasion potential of GC cells. The CHIP-silencing promoted the development and metastasis of GC. The TRAF2-silencing inhibited AGS cell growth, proliferation, migration and invasion, indicating that TRAF2 played an oncogenic role in GC cells. These results were in line with the results from the CHIP-overexpressing AGS cells and were contrast to the results got from the CHIP-silencing AGS cells. A significant negative correlation between the expression of CHIP and TRAF2 was observed in the GC tissues. TRAF2 expression together with tumor diameter was independent prognostic factors in GC patients. Therefore, it was possible that TRAF2 was a substrate of CHIP and CHIP regulated the TRAF2/NF-κB axis, which modulated diverse cellular behaviors of the AGS gastric cancer cells.

## Materials and methods

### Tissue culture

The human gastric cancer cell line AGS was purchased from American Type Culture Collection. Cells were cultured in RPMI-1640 media containing 10% fetal bovine serum (FBS, Gibco, USA), 100 U/ml penicillin, 100 µg/ml streptomycin, and 2 mM glutamine. The pH of the media was adjusted to 7.4 with NaHCO_3_. Cells were maintained in a humidified atmosphere with 5% CO_2_ at 37 °C.

### Cell transfection

The short hairpin RNA (shRNA) specifically targeting the human *CHIP* gene or the human *TRAF2* gene was designed and synthesized from Invitrogen (Beijing, China). The sequences of CHIP-shRNA are 1013–1032: 5′-*AGGCCAAGCACGACAAGTAC*-3′. The sequences of TRAF2-shRNA are 1224–1242: 5′-*GATGTGTCTGCGTATCTAC*-3′. Either the shRNA-CHIP or the shRNA-TRAF2 was subcloned into the pSilencer3.1-H1-neo plasmid (Cat Nr. 5770, Thermo Scientific™, China), which was linearized by restriction endonucleases *Hind*III and *BamH*I. Cells were pre-cultured to 60–80% confluence and transfected using Lipofectamine 2000 (Cat Nr. 12566014, Thermo Scientific™, China) for 6 h according to manufacturer’s protocol. To obtain stably transfected clones, cells were selected in the media containing G418 (400 ng/µl, Cat Nr. E859-5G, Amresco, USA) for 2 weeks.

### Quantitative real-time polymerase chain reaction (qRT-PCR)

Total RNA was isolated using the TRIzol reagent (Tiangen Biotech Co., Ltd., Beijing, China) according to manufacturer’s protocol. RNA yield and purity were determined spectrophotometrically at 260–280 nm by Nanodrop-1000 (Thermo Fisher Scientific, China). cDNA was transcripted using superscript Moloney Murine Leukemia Virus (M-MLV, Cat Nr. 28025013, Promega, China) according to manufacturer’s protocol. The primers of target genes were designed by Primer-BLAST (PubMed) and synthesized from Invitrogen. *β*-*actin* was used as an endogenous control. qRT-PCR was performed using 2 × LC480 SYBR-green IMaster Mix (Cat Nr. 11602920, Roche, China). The PCR conditions were 95 °C for 15 s, 40 cycles of 95 °C for 5 s, 60 °C for 30 s, and 72 °C for 45 s. The average threshold cycle (Ct) for triplets was used to calculate ∆Ct. Relative quantification of mRNA expression was determined by the 2^−ΔΔCt^ method in comparison with internal control. Each experiment was run triplets with a LightCycler 480 System (Roche, China). The sequences of primers are as following: *β*-*Actin*-*R: 5′*-*GCTACGAGCTGCCTGACGG*-*3′*; *β*-*Actin*-*F: 5′*-*TGTTGGC GTACAGGTCTTTGC*-*3′*; *CHIP*-*R: 5′*-*GCCAAGGAGCAGCGGCTGAA*-*3′*; *CHIP*-*F: 5′*-*CTCTCACGCTCCGCGGCAAT*-*3′*; *TRAF2*-*R: 5′*-*GACGTGAAGGCGCACCACGA* -*3′*; *TRAF2*-*F: 5′*-*ACCGTCTCGAGGCAGCCGAT*-*3′*.

### Cell growth assay

Cell growth was monitored with a real-time x-Celligence RTCA instrument (Roche Applied Science, Penzberg, Germany) according to manufacturer’s instructions. 4000 cells per well were seeded in 200 μl media containing 10% FBS in an E-plate. The E-Plates were continuously monitored on a RTCA system for 72 h at 37 °C with 5% CO_2_. The impedance was measured as “cell index”. Data were collected and analyzed by RTCA software 1.2.

### Colony formation assay

Soft agar assays were performed as follows. Briefly, 10,000 cells were mixed with soft agar (0.3%), and subsequently layered on solidified agar (0.6%). After 14 days’ incubation, colonies larger than 75 μm in diameter or containing more than 50 cells were counted as a positive colony. Colonies were visualized using crystal violet, photographed, and counted.

### Ki-67 assay

Cells were cultured on cover slides for 24, 48, and 72 h in a humidified incubator at 37 °C and 5% CO_2_. Slides were fixed in 4% paraformaldehyde for 30 min at 4 °C and treated with phosphate-buffered saline (PBS) containing 1% Triton for 10 min. Slides were treated with 3% hydrogen peroxide for 10 min to reduce endogenous peroxidase activity. The slides were incubated with an antibody (Ab) against Ki-67 (1:100 dilutions, Cat Nr. M7240, Dako, China) for 1 h at room temperature (RT). A secondary Ab (Cat Nr. GK500705, rabbit anti-mouse IgA-B, GTvision III) was applied to the sections and incubated for 30 min. Finally, 3, 3-diaminobenzine (DAB) was used to visualize the immune-reactive products. Results were evaluated by a light system microscope (IX71, Olympus, Japan). The Ki-67 expression was detected in the nuclei of cells. The representative visual fields (400× magnifications) were chosen to calculate the percentage of positive cells over the total tumor cells by two independent pathologists. The results were evaluated quantitatively and divided into four groups, negative; +: < 30% of tumor cells were positive; ++: 30–60% of tumor cells were positive; and +++: > 60% of tumor cells were positive.

### Cell counting kit-8 (CCK-8) assay

To assess the cell proliferation, a CCK-8 assay (Cat Nr. CK04, Dojindo Molecular Technologies, Inc., Kumamoto, Japan) was performed. Briefly, 5000 cells/well were seeded into a 96-well plate and measured at the indicated times according to the manufacturer’s instructions. At the end of each experiment, 10 μl CCK-8 reagents were added into each well and incubated at 37 °C for 2 h. The absorbance of 450 nm was measured using a microplate reader (Elx800, BioTek) to calculate cell growth rates. Each experiment was performed in triplicate and repeated three times.

### Cell viability assay

Cell viability was assessed using a CellTiter-Glo^®^ luminescent cell viability assay kit (Cat Nr. G755A, Promega Co., Ltd., USA). Briefly, 4000 cells were seeded in 96-well plates and incubate at 37 °C for 24, 48, and 72 h. 10 µl reagents were added to each well. Absorbance was measured at 570 nm using a spectrophotometer microplate reader (Flx800, BioTek, USA). Each experiment was repeated in triplicate.

### TdT-mediated dUTP Nick-End Labeling (TUNEL) assay

According to the manufacturer’s instructions (Cat Nr. 11684795910, Roche, China), cells were cultured on cover slides for 24, 48, and 72 h in a humidified incubator at 37 °C and 5% CO_2_. The slides were fixed in 4% paraformaldehyde for 30 min at 4 °C and then treated with 1% Triton PBS solution for 10 min. The slides were treated with 3% hydrogen peroxide for 10 min to reduce endogenous peroxidase activity. Then, the sliders were added with 50 µl working-strength TdT solutions at 37 °C for 1 h. The signals of TUNEL were converted using peroxidase (POD) at 37 °C for 30 min and the sliders were treated with DAB for 3 min at RT. Results were examined by a light system microscope (IX71, Olympus, Japan).

### Apoptosis assay

The apoptosis assay was performed with an Alexa Fluor^®^ 488 Annexin V/Dead Cell Apoptosis Kit (Cat Nr. V13241, Invitrogen, China) following manufacturer’s instruction. Cells were washed with PBS, and stained with Annexin V alone or together with propidium iodide (PI, Cat Nr. P4170, Sigma, Germany) for 15 min at RT in the dark. Cell apoptosis was examined by a FACSCalibur™ cytometer (BD Biosciences, USA) and analyzed by CellQuestPro software.

### Cell cycle analysis

Cells were trypsinized, washed with PBS, and then incubated with 50 µg/ml PI and 20 mg/ml RNase A (Cat Nr. 12091-021, Invitrogen, USA) for 20 min at 37 °C in the dark according to manufacturer’s instructions. Cell cycle was measured by a FACSCalibur™ cytometer. The distributions of cells in the G_0_–G_1_, S, and G_2_-M phases were analyzed by ModFit LT software. All samples were run in triplicate in at least three independent experiments.

### Western blotting assay

Cells were lysated with a modified RIPA lysis buffer in the presence of protease inhibitor (complete Tablets Mini EDTA-free, Roche Diagnostics, Basel, Switzerland). Protein concentrations were measured with a BCA Protein Assay Kit (Cat Nr. 23225, HyClone-Pierce, South Logan, USA). A total of 10 μg protein extracts was electrophoresed on 8–10% sodium dodecyl sulfate (SDS)-PAGE gels and semi-electrically transferred into nitrocellulose membranes. After blocking in 5% skim milk for 1 h at RT, the membranes were incubated with the indicated primary Abs overnight at 4 °C. After several washes, the membranes were incubated with the secondary Abs for 1 h at RT. Proteins were detected and scanned by an Odyssey^®^ infrared imaging system (LI-COR Biosciences, Lincoln, NE, USA). Band density was normalized to Actin expression. Abs against TRAF2 (C-20, sc-876), RelA/p65 (C-20, sc-372), RelB (C-19, sc-226), c-Rel (N, sc-70), NF-κB p105/50 (H-119, sc-7178), NF-κB p100/52 (K-27, sc-298), Bcl-2 (C-2, sc-7382), Lamin A/C (H-110, sc-20681), and uPA (H-140, sc-14019) were purchased from Santa Cruz Biotechnology. Abs against CHIP (#2080), Cyclin D1 (#2978), Cyclin D3 (#2936), cyclin-dependent kinase 4 (CDK4, #12790), cyclin-dependent kinase 6 (CDK6, #3136), ERK1/2 (#4695), p-ERK1/2 (#4370S), AKT (#4691), p-AKT-308 (#2965), p-AKT-473 (#4060), BIM (#2933), Integrin β1 (#9699), MMP2 (D8N9Y), MMP9 (D603H), and p53 (#2527) were purchased from Cell Signaling Technology, Inc. Ab against Actin (AT0001) was purchased from Abgent (Suzhou, China). IRDye 680CW (#926-32222) and IRDye 800CW (#926-32210) secondary Abs were purchased from LI-COR Biosciences.

### Scratch healing assay

Scratch was created using a 200-μl sterile pipette tip, and washed with PBS. The wound closure was photographed with a light System microscope (IX71, Olympus, Japan) at 0, 24, and 48 h. The gap distance is used to measure the wound healing ability/migration velocity. Experiments were repeated three times.

### Cell migration and invasion assay using an x-Celligence system

Cell migration was tested using the real-time x-Celligence system with CIM-plate 16. Lower chambers were added with RPMI-1640 media (180 µl) supplemented with 10% FCS, used as a chemo-attractant. Upper chambers were filled with serum-free media (100 μl/well). Then the plate was placed in humidified incubator at 37 °C and 5% CO_2_ for 1 h. After recording background measurements, the cells were seeded in the serum-free media and added into the upper chamber at 40,000 cells in 100 μl per well. The plate was incubated for 30 min at RT and assembled on a RTCA DP analyzer. Data was gathered at 5-min intervals for 24 h at 37 °C in a 5% CO_2_ humidified atmosphere. The data was analyzed using RTCA 1.2 software. Cell invasion assay was also monitored with the xCelligence system. The upper chamber was pre-coated with Matrigel (Cat Nr. 356234, BD Biosciences, USA) for 6 h. Cell invasion through Matrigel towards the lower chamber was continuously monitored by the x-Celligence system, and data were collected and analyzed by RTCA software 1.2.

### Cell migration and invasion assay using a Transwell system

The invasive potential of cells was evaluated with Transwell chambers (Falcon cell culture inserts, 24-well format, 8 µm pore size, BD Biosciences, USA). A total of 60,000 cells in 250 µl serum-free media were introduced into upper chambers coated with 50 µl Matrigel. Complete media containing 10% FCS served as a chemo-attractant was introduced into lower chambers. Cells were then incubated for 24 h at 37 °C. The cells on the upper surface of the filter were removed by cotton swabs. Invaded cells were fixed in methanol and stained with 0.1% crystal violet. Cells in five visual fields of each well were counted and photographed under a microscope. Migration assays were performed using the same procedure, except that the insert chambers were not coated with Matrigel.

### NF-κB DNA-binding capability

The DNA-binding capacity of NF-κB was measured in the nuclear extracts of cells using a TransAM NF-κB kit (Cat Nr. 43296, Active Motif, Carlsbad, CA, USA) according to manufacturer’s instructions. Briefly, nuclear extracts (5 μg) from each sample were incubated for 1 h in 96-well plates, upon which an oligonucleotide containing the NF-κB consensus binding site (5′-*GGGACTTTCC*-3′) was immobilized. The subunits of NF-κB contained in the extracts are able to specifically bind to this nucleotide. Then individual primary NF-κB Abs (1:1000) were added for 1 h, and subsequently peroxidase-conjugated secondary Ab (1:1000) was incubated for 1 h at RT. Stop solution (100 μl) was added to each well and the color of the sample well was changed from blue to yellow. After colorimetric reaction, optical density (OD) was read at 450 nm with a microplate reader (Elx800, BioTek).

### Carboxyfluorescein diacetate succinimidyl ester (CFSE) assay

Cells were detached and stained with 5 μM CFSE (Cat Nr. C-1311, Molecular Probes, China) in PBS for 10 min at 37 °C. The reaction was stopped by the addition of an equal volume of FBS, followed by a 5-min’ incubation at RT. Followed by washing with PBS for two times, the CFSE-labeled target cells were re-suspended in assay medium and either directly used or cultured for 24, 48, and 72 h at 37 °C and 5% CO_2_. The fluorescence intensity was measured using a FACSCalibur™ cytometer.

### Clinical samples

The commercial GC tissue microarray was purchased from Shanghai Outdo Biotech Company from the National Human Genetic Resources Sharing Service Platform (2005DKA21300). Use of patient samples and clinical data in this study was approved by the Ethics Committee of Shanghai Outdo Biotech Company. The expression of CHIP and TRAF2 protein were evaluated in 100 paired GC and non-neoplastic tissues. Patients’ follow-up information was obtained from 2006 to 2014.

### Immunohistochemistry (IHC)

The tissue slides were de-paraffinized at 60 °C for 1 h, then de-waxed in xylene for 3 × 10 min and rehydrated gradually in 100%, 100%, 90%, 80%, and 70% ethanol for 3 min each. Antigen retrieval was performed in a pressure cooker containing citrate buffer for 5 min. Slides were treated with 3% hydrogen peroxide for 10 min to reduce endogenous peroxidase activity. Followed by washing with PBS, the slides were incubated with 10% FBS for 30 min at 37 °C. CHIP (Cat Nr. AP6413B, Abgent, San Diego, CA, USA) and TRAF2 primary Abs (Cat Nr. C-20, sc-876, Santa Cruz Biotechnology) were applied overnight at 4 °C. For negative control, the immune-staining processes were performed by using PBS as a substitute for the primary Ab. The antigen–antibody complex was detected by using diaminobenzidine (DAB) substrate. Slides were then counter-stained with haematoxylin, dehydrated in a graded series of ethanol and xylol. Images were captured using a microscope (BX51, Olympus, Japan). Two experienced pathologists analyzed the IHC results. Visual fields (×400 magnifications) were chosen to calculate the percentage of positively stained cells over the total number of tumor cells. The staining proportion of the positive cells was divided into four groups: negative, 0 positive cells found; +: < 30% of tumor cells observed; ++: 30–60% of tumor cells were immune-positive; and +++: > 60% of tumor cells were immune-positive. Cases with proportion scores of – and + were included in the CHIP-low expression group, while those with proportion scores of ++ and +++ were included in the CHIP high expression group for all of the analysis. Data sets were combined after the completion of scoring and analyzed by Fisher’s exact *t* test.

### Statistical analysis

The data were expressed as mean ± standard deviation (SD) of at least three separate experiments and were analyzed by Student’s *t*-test between two groups. The significance of correlations between clinic-pathological characteristics and expression of CHIP and TRAF2 were analyzed respectively by Student’s *t*-test and Pearson’s χ^2^ test. The survival curves were analyzed using Kaplan–Meier analysis. The association between CHIP and TRAF2 expression was analyzed by Spearman’s test (r; *p*-value). Multivariate analyses were performed using the Cox proportional hazards model. All statistical analyses were two-sided and performed by SPSS 24.0 software. Figures was created using GraphPad Prism 5.0. *p *< 0.05 was considered as statistically significant. * for *p *< 0.05, ** for *p *< 0.01, *** for *p *< 0.001.

## Results

### CHIP affects AGS cell growth

The *CHIP* expression at mRNA level was decreased about 2.93-fold (*p *< 0.001) in the siCHIP cells (AGS cells transfected with the CHIP-shRNA plasmid) compared to that in the sictrl cells (AGS cells transfected with the control-shRNA plasmid) detected by qRT-PCR (Additional file 1: Fig. S1a). In line with the decreased mRNA level, the CHIP expression at protein level was also evidently reduced by Western blotting analysis (Additional file 1: Fig. S1b). Therefore, a successful RNA-interference targeting the *CHIP* gene was established in the AGS gastric cancer cells.

**Fig. 1:**
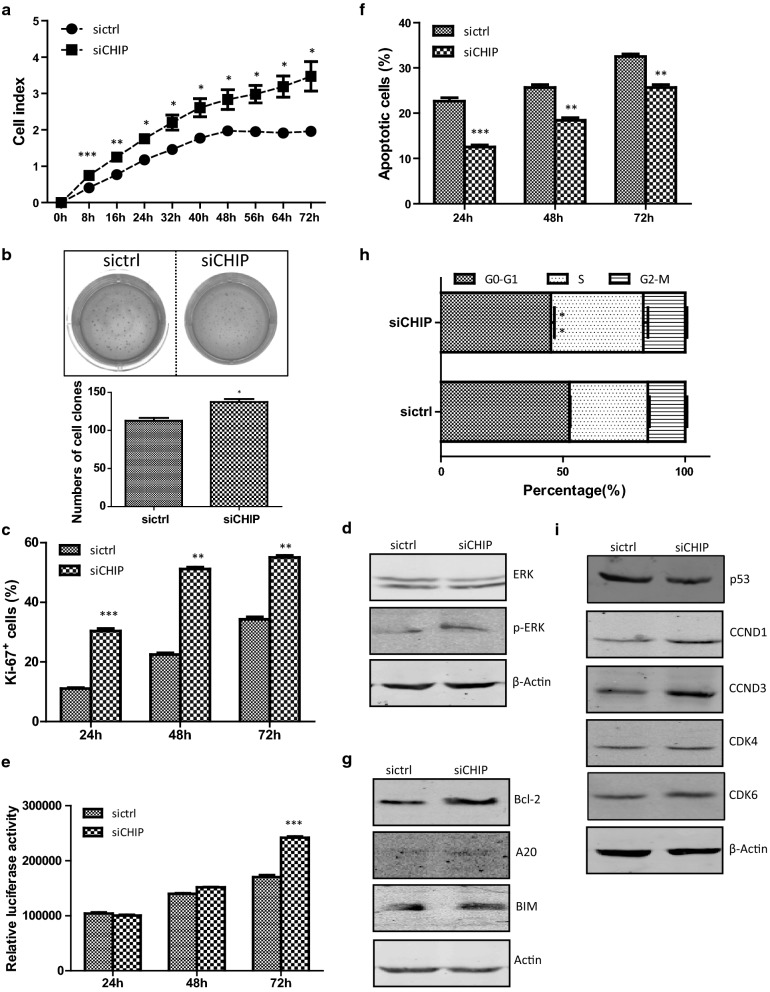
CHIP-silencing enhances AGS cell growth. **a** The cell growth curves of the sictrl and siCHIP cells were detected by an x-Celligence system. E-plates were plated with 4000 cells/well and cell growth was continuously monitored for 72 h. **b** The anchorage-independent growth of the sictrl and siCHIP cells were detected using soft agar. 24-well plates were plated with 10,000 cells/well. Colonies were counted after 14 days’ incubation. **c** The bar chart represents the frequencies of Ki-67^+^ cells of the two established cell lines. **d** Western blotting analysis of the protein expression of total ERK1/2 and p-ERK1/2 in siCHIP and sictrl cells. Actin was used as an internal control. **e** Cell viability assay. 96-well plate was plated with 4000 cells/well. OD570 was measured by means of spectrophotometer microplate reader at 24, 48 and 72 h. **f** A TUNEL assay was carried out to quantitatively analyze the apoptotic cells. The bar chart represents the percentages of apoptotic cells. **g** Western blotting analysis of apoptosis-associated proteins. Actin was used as an internal control. **h** Cell cycle assay was determined by flow cytometry analysis using PI-staining. The histograms represent the percentages of cells in G_0_–G_1_, S, and G_2_-M phases. **i** Western blotting analysis of cell cycle-related proteins. Actin was used as an internal control

The x-Celligence monitoring system was used to investigate whether the CHIP-silencing played a role on AGS cell growth. As shown in the Fig. 1a, the cell index was 0.40 ± 0.01 for the sictrl cells, and 0.74 ± 0.03 for the siCHIP cells at 8 h (*p *< 0.001). The cell index of the siCHIP cells was continuously higher than that of the sictrl cells at all time points during the 72 h’s culture, and there were statistically significant difference between the two cell lines after 8 h (*p *< 0.01 at 16 h time point, *p *< 0.05 from 24 h to 72 h). The clearly separated growth curves revealed that the CHIP-silencing markedly promoted AGS cell growth. Anchorage-independent growth of AGS cells was examined by a soft-agar colony formation assay. The number of colonies was 112.67 ± 6.43 in the sictrl cells, and 137.33 ± 7.02 in the siCHIP cells (*p *< 0.05, Fig. 1b). More colonies formed from siCHIP cells than sictrl cells in soft agar. The CHIP-silencing in the AGS cells played an oncogenic role evidenced by the appearance of capable of anchorage-independent growth.

A Ki-67 cell proliferation assay was performed to investigate whether CHIP affected the proliferation capability of AGS cells. The frequencies of Ki-67^+^ cells were 11.09% ± 0.25%, 22.51% ± 1.02%, and 34.35% ± 1.83% in the sictrl group, while 30.36% ± 1.93%, 51.16% ± 1.24%, and 55.08% ± 1.02% in the siCHIP group at 24, 48, and 72 h, respectively (Fig. 1c). There were statistically significant differences between the two cell lines at 24 h (*p *< 0.001), 48 h and 72 h (*p *< 0.01). The expression of total protein kinase B (PKB, also known as AKT) and the phosphorylation of AKT (p-AKT^473^ and p-AKT^308^) were unchanged in the siCHIP cells. A negative regulator of the AKT signaling, phosphate and tension homology deleted on chromosome ten (PTEN), was also unchanged (Additional file 1: Fig. S2). The expression level of extracellular regulated protein kinases (ERK) was similar between the sictrl and siCHIP cells. However, the CHIP-silencing in the AGS cells led to significant induction of p-ERK, indicating of the activation of the ERK signaling (Fig. 1d). Therefore, the CHIP-silencing significantly enhanced the AGS cell proliferation capability likely due to the induced phosphorylation of ERK.

Cell viability assay was performed to determine the number of viable cells. The OD values of relative luciferase activities were 104168.50 ± 4076.12, 139931.00 ± 1784.38, and 170422.00 ± 6160.63 in the sictrl cells, while 100369.00 ± 2421.21, 151755.00 ± 620.89, and 241880.50 ± 4239.58 in the siCHIP cells at 24, 48, and 72 h, respectively (Fig. 1e). There was a statistically difference between the two cell lines at 72 h (*p *< 0.001), indicating that the siCHIP cells survives better than sictrl cells. A TUNEL assay was carried out to investigate the role of CHIP in apoptosis. Spontaneous apoptosis, in a time-dependent manner, was identified in both the sictrl and the siCHIP cells. The percentages of apoptotic cells in the sictrl group were 22.67% ± 1.28%, 25.67% ± 1.07%, and 32.50% ± 0.96%, while those in the siCHIP group were 12.54% ± 0.70%, 18.41% ± 1.20%, and 25.66 ± 1.08% at 24, 48, and 72 h, respectively. There were statistically significant differences between the two cell lines at 24 h (*p *< 0.001), 48 h and 72 h (*p *< 0.01), indicating that the CHIP-silencing suppressed the apoptosis of AGS cells (Fig. 1f). The expression of the B-cell lymphoma-2 (Bcl-2), an important anti-apoptotic molecule, was increased in the siCHIP cells. Other apoptosis-associated proteins such as A20 and Bcl-2 interacting mediator of cell death (BIM) in the siCHIP cells stayed unchanged compared to that in the sictrl cells (Fig. 1g). Therefore, the CHIP-silencing significantly inhibited the AGS apoptosis due to increased expression of Bcl-2.

The effects of CHIP on cell cycle and cellular DNA content were examined by flow cytometry. The distributions of G_0_–G_1_, S, and G_2_-M phases in the sictrl cells were 52.62% ± 0.61%, 32.03% ± 1.23%, and 15.34% ± 1.15%, while those in the siCHIP cells were 45.07% ± 2.43%, 37.74% ± 3.39%, and 17.19% ± 1.17%. Cell cycle progression of the siCHIP cells was notably promoted the G1/S phase transition and entry into the S-phase (*p *< 0.01, Fig. 1h). The expression Cyclin D1 and Cyclin D3 was increased in the siCHIP cells, while the expression of p53 was decreased (Fig. 1i). The expression levels of CDK4 and CDK6 in the siCHIP cells stayed unchanged compared to that in the sictrl cells. Collectively, the CHIP-silencing enhanced AGS cell growth, owing to induced proliferative activity, deduced apoptosis, and induced G1/S phase transition.

### CHIP affects migration and invasion abilities

**Fig. 2:**
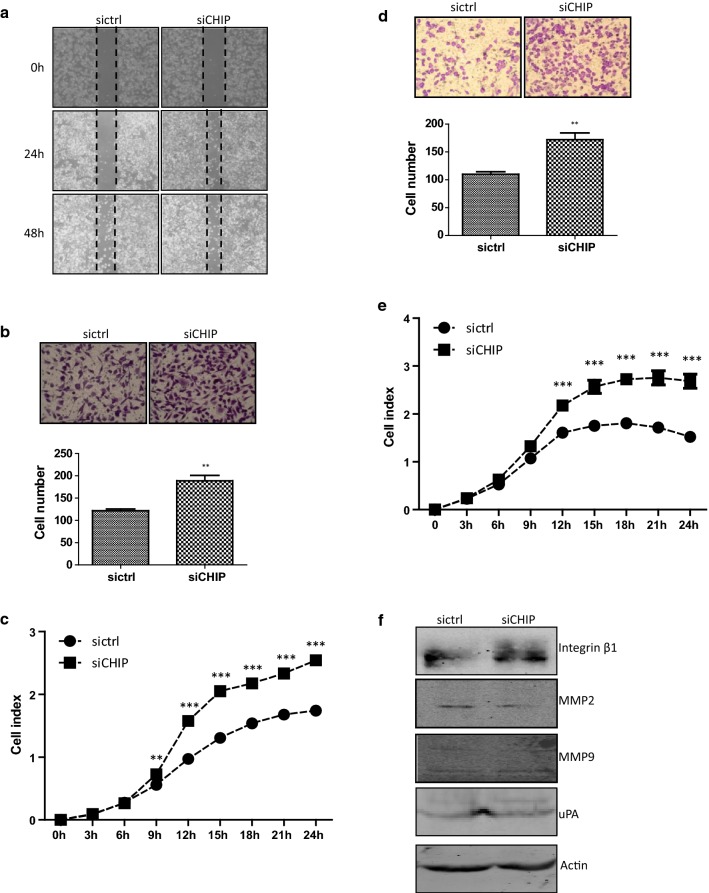
CHIP affects the migration and invasion abilities of AGS cells. **a** The migration ability of the cells was detected by the scratch healing assay at 24 h and 48 h. **b** Representative images of the Transwell migration assay. The numbers of migrated cells were calculated. Cells were fixed, stained, and photographed. Bar chart represents mean ± SD. All images were representative of at least three independent experiments with similar findings. **c** The migration curves of the sictrl and siCHIP cells were detected using the real-time x-Celligence system. **d** Representative images of the Transwell invasion assay. The sictrl and siCHIP cells were allowed to migrate through the Transwell inserts pre-coated with Matrigel (1:40 dilutions) for 24 h. The numbers of invasive cells were calculated. Cells were fixed, stained, and photographed. Each bar represents mean ± SD. All images were representative of at least three independent experiments with similar findings. **e** The invasion ability of the two established cell lines was detected by the real-time xCelligence monitoring system using Matrigel (1:40 dilutions)-coated CIM-plates. **f** Western blotting analysis of protein expression of Integrin-β1, uPA, MMP2, and MMP9. Actin was used as an internal control

A scratch assay was carried out to examine whether CHIP regulated the migration ability of AGS gastric cancer cells. The migration velocity was estimated by migratory distance. Representative images were captured under microscope at 0, 24, and 48 h, respectively. The siCHIP cells migrated from the edge of the scratch toward the scratch centre much quicker than the sictrl cells (Fig. 2a). Transwell inserts were used to examine the migration ability of AGS cells. Cells which had migrated out of the inserts were counted after 24 h. The average numbers of migratory siCHIP cells were 188.67 ± 21.01, much more than that of the sictrl cells, 121.67 ± 6.66 (*p *< 0.01, Fig. 2b). Effects of CHIP on the migration ability were also examined by the real-time x-Celligence system using CIM-plates. As shown in Fig. 2c, the siCHIP cells migrated much quicker than the sictrl cells. The cell index was 0.56 ± 0.03 in the sictrl cells, and 0.73 ± 0.02 in the siCHIP cells at 9 h time point. There were statistically significant differences between the two established cell lines from 9 to 24 h (*p *< 0.01 at 9 h, *p *< 0.001 at 12, 15, 18, 21, and 24 h). Taken together, the CHIP-silencing increased the migration ability of the AGS cells.

Transwell insert, pre-coated with Matrigel (dilution at 1:40), was used to investigate the invasion ability of AGS cells. The numbers of invaded siCHIP cells were 172.33 ± 20.50, evidently more than that of the invaded sictrl cells, 110.00 ± 7.80 (*p *< 0.01, Fig. 2d). The invasion ability was detected also used the real-time x-Celligence system with CIM-plate pre-coated with Matrigel. As shown in Fig. 2e, the cell index was 1.61 ± 0.041 in the sictrl cells, and 2.18 ± 0.11 in the siCHIP cells at 12 h time point. The siCHIP cells invaded through the Matrigel faster than the sictrl cells. There were statistically differences between the two established cell lines from 12 to 24 h (*p *< 0.001 at 12, 15, 18, 21, and 24 h). The CHIP-silencing markedly promoted the cell invasion capacity of AGS cells. A variety of molecules are involved in regulating cellular migration and invasion of cancer cells. The increased Integrin β1 expression was observed while the expression of uPA, matrix metallopeptidase 2 (MMP2), and matrix metallopeptidase 9 (MMP9) was unchanged in the siCHIP cells (Fig. 2f). These results suggested that CHIP suppressed the AGS cell migration and invasion abilities, likely by regulating the expression of Integrin β-1.

### CHIP regulates NF-κB signaling pathway through TRAF2

**Fig. 3:**
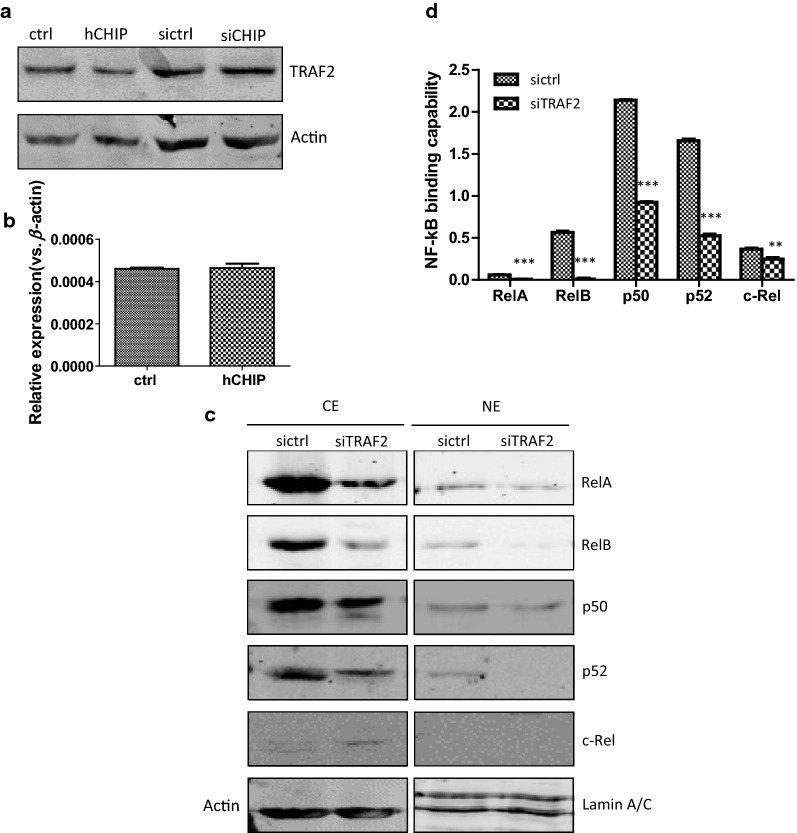
CHIP regulates NF-κB signaling pathway through TRAF2. **a** The protein expression of TRAF2 in the ctrl, hCHIP, siCHIP, and sictrl cell lines was analyzed by Western blotting analysis. Actin was used as an internal control. **b** The mRNA expression of *TRAF2* in the ctrl and hCHIP cell lines. *β*-*actin* normalized gene expression, measured in triplicates was displayed. **c** The protein expression of NF-κB subunits were examined by Western blotting analysis. Protein expression in CE and NE was normalized against Actin or Lamin A/C, respectively. **d** The DNA-binding activity of NE was detected and quantified using a TransAM NF-κB family transcription factor assay kit

We have previously analyzed the biological functions of CHIP overexpression in the AGS gastric cancer cells. CHIP overexpression caused impaired tumor growth and inhibited the migration and invasion abilities of the AGS cells. RelA, the NF-κB subunit, was negatively regulated by CHIP, likely owing to the TRAF2 reduction [13]. TRAF2 positively regulates the activity of the canonical and non-canonical NF-κB signal pathway. In line with our previous data, TRAF2 was markedly decreased in the CHIP-overexpressing cells at protein level, but not at mRNA level (Fig. 3a, b).

To dissect whether the biological function of CHIP in the AGS gastric cancer cells was due to its target TRAF2, an RNA-interference targeting the *TRAF2* gene was established in the AGS gastric cancer cells. As shown in Additional file 1: Fig. S3a, the *TRAF2* expression at mRNA level was decreased about 1.87-fold (*p *< 0.001) in the siTRAF2 cells (AGS cells transfected with the TRAF2-shRNA plasmid) compared to that in the sictrl cells (AGS cells transfected with the control-shRNA plasmid) examined by qRT-PCR. In line with the decreased mRNA level, the TRAF2 expression at protein level was reduced detected by Western blotting analysis (Additional file 1: Fig. S3b).

The expressions of all subunits of the NF-κB family were clearly decreased in both the cytoplasmic (CE) and nuclear extracts (NE) of the siTRAF2 cells compared to that of the sictrl cells (Fig. 3c). The NF-κB DNA-binding activity was measured with a sensitive colorimetric assay using a specific oligonucleotide probe for NF-κB. To investigate the effects of the TRAF2-silencing on NF-κB DNA-binding capability, an ELISA-based assay was used. Compared to that in the sictrl cells, the average RelA, RelB, p50, and p52 DNA-binding capabilities in NE of the siTRAF2 cells were markedly reduced (*p *< 0.001, Fig. 3d). The c-Rel DNA-binding capability was moderately reduced (*p *< 0.01).

### TRAF2 affects AGS cell growth

**Fig. 4:**
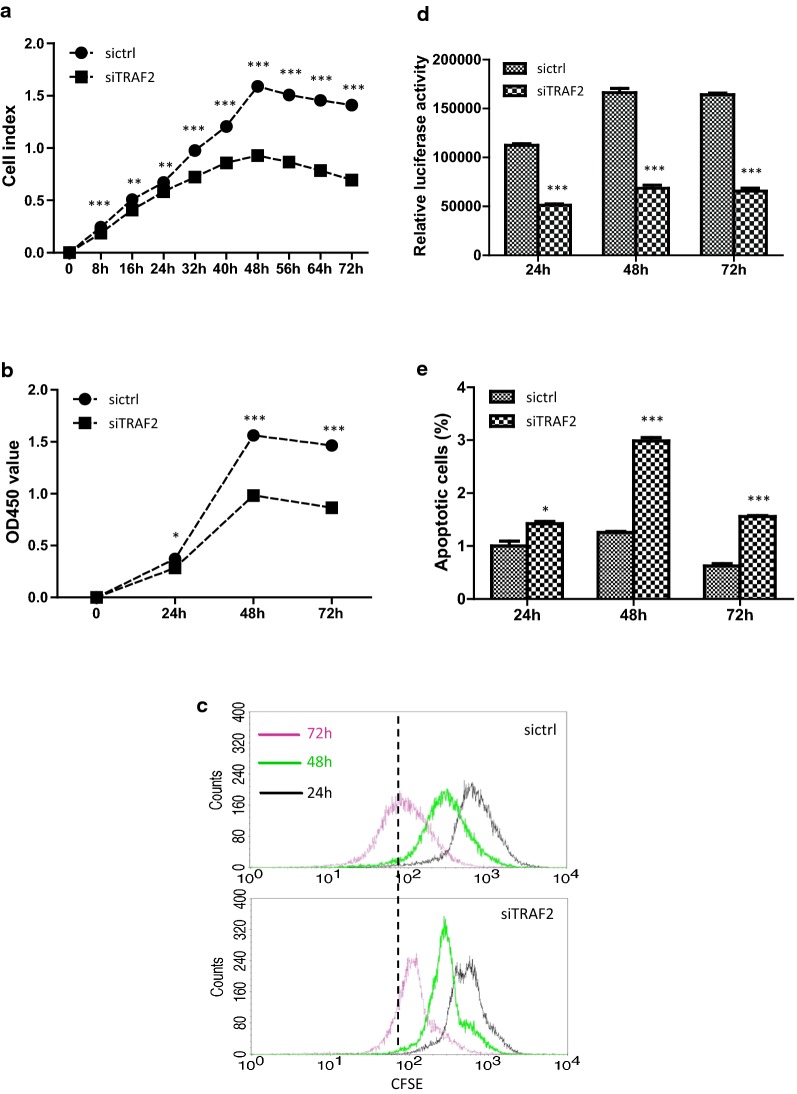
TRAF2-silencing inhibited the cell growth of AGS cells. **a** The cell growth curves of the sictrl and siTRAF2 cells were detected using the x-Celligence system. E-plate was seeded with 4000 cells/well and cell growth was continuously monitored for 72 h. **b** Cell proliferation of the siTRAF2 and sictrl cells was determined by the CCK-8 assay. 96-well plate was seeded with 5000 cells/well. OD450 was measured by means of spectrophotometer microplate reader. **c** The CFSE proliferation assay was examined by flow cytometry analysis at 24, 48 and 72 h. **d** Cell viability assay. 96-well plate was plated with 4000 cells/well. OD570 was measured by means of spectrophotometer microplate reader at 24, 48 and 72 h. **e** The apoptosis of the sictrl and siTRAF2 cells was determined by the Annexin V/PI staining. The bar chart represents the percentages of apoptotic cells of the two established cell lines. The mean ± SD from three individual experiments were displayed

The real-time x-Celligence system was used to explore whether the TRAF2-silencing influenced AGS cell growth. As shown in Fig. 4a, the cell index was 0.24 ± 0.01 in the sictrl cells, and 0.19 ± 0.01 in the siTRAF2 cells at 8 h time point. The cell index of the siTRAF2 cells was much lower than that of the sictrl cells at all time points during 72 h’s continuous monitoring, and there were statistically difference between the two cell lines (*p *< 0.001 at 8 h, *p *< 0.01 at 16 h and 24 h, and *p *< 0.001 from 32 to 72 h). Cell growth curves clearly revealed that the TRAF2-silencing inhibited AGS cell growth.

Cell proliferation was examined by the CCK-8 assay (Fig. 4b). The OD450 values of the sictrl cells were 0.37 ± 0.05, 1.56 ± 0.11, and 1.46 ± 0.10, while those of the siTRAF2 cells were 0.28 ± 0.03, 0.98 ± 0.10, and 0.86 ± 0.08 at 24, 48 and 72 h, respectively. The OD450 values were clearly declined in the siTRAF2 cells, and there were statistically significant differences between the two cell lines at 24 h (*p *< 0.05), 48 h and 72 h (*p *< 0.001). The cellular proliferation was also detected by a CFSE assay. As shown in Fig. 4c, the fluorescence intensities of CFSE were attenuated in both established cell lines in a time-dependent manner. However, the siTRAF2 cells proliferated at a markedly slower rate than the sictrl cells at 72 h time point. Therefore, the TRAF2-silencing inhibited the proliferation ability of the AGS cells.

The relative luciferase activities in the cell viability assay were 112347.30 ± 3341.51, 166221.00 ± 8858.15, and 164306.00 ± 3278.85 in the sictrl cells, while 51296.00 ± 2631.062, 68389.25 ± 6703.21, and 65559.50 ± 6339.22 in the siTRAF2 cells at 24, 48, and 72 h, respectively (Fig. 4d). There were statistically significant differences between the two cell lines at 24, 48, and 72 h (all *p *< 0.001), suggesting that the siTRAF2 cells died more than the sictrl cells. Apoptosis was further measured using Annexin V/PI-staining. The rates of spontaneous apoptosis, indicated by double-positive for Annexin V and PI, were 1.00%, 1.26%, and 2.98% in the sictrl cells, and 1.42%, 2.98%, and 1.56% in the siTRAF2 cells at 24, 48, and 72 h, respectively. There were statistically significant differences between the two cell lines at 24 h (*p *< 0.05), 48 h and 72 h (*p *< 0.001), indicating that the TRAF2-silencing promoted the apoptosis of AGS cells (Fig. 4e). Thus, the TRAF2-silencing suppressed the AGS cell growth, the defected cell proliferation and enhanced apoptosis were involved in.

### TRAF2 affects the migration and invasion abilities of AGS cells

**Fig. 5:**
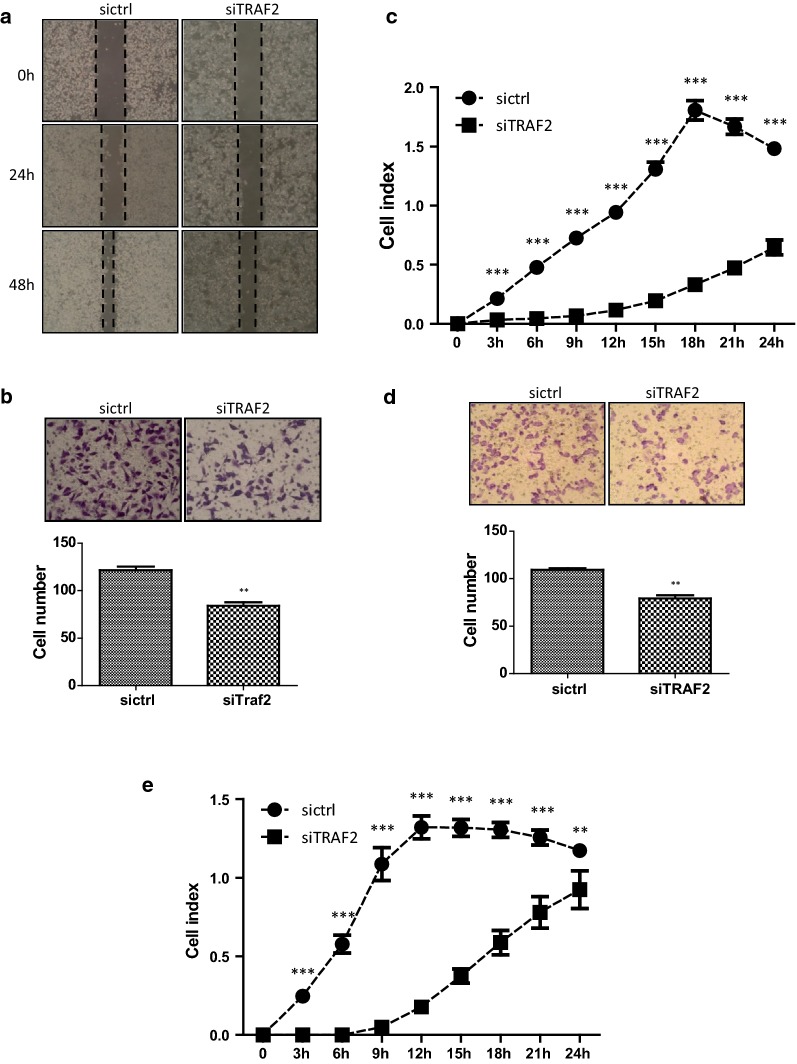
TRAF2-silencing inhibited the migration and invasion of AGS cells. **a** The migration ability of the cells was detected by the scratch healing assay at 24 h and 48 h. **b** Representative images of the Transwell migration assay. The numbers of migrated cells were calculated. Cells were fixed, stained, and photographed. Bar chart represents mean ± SD. All images were representative of three independent experiments. **c** The migration abilities of the sictrl and siTRAF2 cells were detected using the real-time x-Celligence system. **d** Representative images of the Transwell invasion assay. The sictrl and siTRAF2 cells were allowed to migrate through the Transwell inserts pre-coated with Matrigel (1:40 dilutions) for 24 h. The numbers of invasive cells were calculated. Cells were fixed, stained, and photographed. Each bar represents mean ± SD. All images were representative of at least three independent experiments. **e** The invasion ability of the sictrl and siTRAF2 cell lines was detected by the real-time xCelligence system using Matrigel (1:40 dilutions)-coated CIM-plates

The scratch assay was presented in Fig. 5a. According to the representative photos taken at 0, 24, and 48 h, the siTRAF2 cells migrated from the scratch edge to the scratch centre a lot slower than the sictrl cells. Transwell inserts were performed to study the function of TRAF2 on the migration ability of AGS cells. Cells which had migrated the inserts were calculated and photographed after 24 h. The average numbers of the migratory siTRAF2 cells were 84.00 ± 6.56, fewer than that of the sictrl cells, 121.67 ± 6.66 (*p *< 0.01, Fig. 5b). Effects of TRAF2 on the migration ability of AGS cells were also checked by the x-Celligence monitoring system using CIM-plates. There were statistically significant differences between the two cell lines from 3 h to 24 h (all *p *< 0.001, Fig. 5c). The clearly separated migration curves suggested that the TRAF2-silencing inhibited cell migration.

Transwell insert, pre-coated with Matrigel (dilution at 1:40), was used to investigate whether TRAF2 influenced the invasion capability of AGS cells. The numbers of invaded siTRAF2 cells were 79.00 ± 6.25, less than that of the invaded sictrl cells, 109.33 ± 3.06 (*p *< 0.01, Fig. 5d). The invasion capability was also identified used the x-Celligence system with CIM-plate pre-coated with Matrigel. As shown in Fig. 5e, the siTRAF2 cells invaded through the Matrigel greatly slower than the sictrl cells. There were statistically differences between the two established cell lines from 3 to 24 h (3–21 h *p *< 0.001, *p *< 0.01 at 24 h). Together, the TRAF2-silencing markedly attenuated the cell migration and invasion capacity of AGS cells.

### Clinical significance of CHIP and TRAF2 expression in GC

**Fig. 6:**
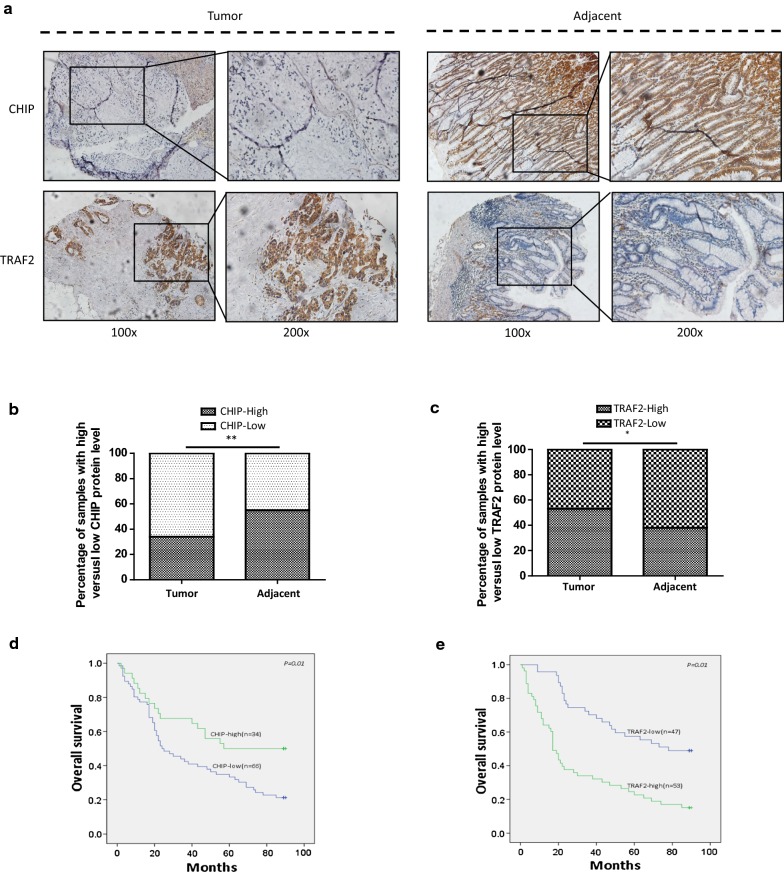
Clinical significance of CHIP and TRAF2 expression in GC patients. **a** Representative images of CHIP and TRAF2 expression in GC and adjacent non-neoplastic tissues were detected by IHC. Tumor represents GC tissues; Adjacent represents the non-neoplastic tissues; magnification 100× and 200×). **b** The stacked bars indicate the percentages of GC samples with high or low CHIP expression levels relative to the total number of tissues. **c** The stacked bars indicate the percentages of GC samples with high or low TRAF2 expression levels relative to the total number of tissues. **d** Kaplan–Meier curves that depict the 5-year OS according to the CHIP expression in patients with GC. CHIP-low represented the CHIP low expression group; CHIP-high represented the CHIP high expression group. The difference of OS between the two groups was determined using a log-rank test. **e** Kaplan–Meier curves that depict the 5-year OS according to the TRAF2 expression in patients with GC. TRAF2-low represented the TRAF2 low expression group; TRAF2-high represented the TRAF2 high expression group

A tissue microarray of 100 GC patients with paired adjacent counterparts was evaluated for the expression of CHIP and TRAF2 by IHC analyses. Representative images of CHIP and TRAF2 expression in GC and adjacent non-neoplastic tissues were shown in Fig. 6a. CHIP was predominantly expressed on the membrane and in the cytoplasm of the adjacent non-neoplastic cells compared to that in the neoplastic parts. In contrast, TRAF2 was mostly detected on the membrane and in the cytoplasm of the GC cells compared to that in the adjacent non-neoplastic parts.

Increased CHIP expression (CHIP-high) was found in 34% (34/100) of GC tissues, whilst CHIP-high was 55% (55/100) in the paired adjacent non-neoplastic tissues. CHIP expression was greatly lower than that in the adjacent normal gastric mucosa, and there was a statistically significant difference (*p *< 0.01) between the GC tissues and the adjacent normal tissues (Fig. 6b). Interestingly, high TRAF2 expression (TRAF2-high) was found in 53% (53/100) of GC samples, while TRAF2-high was only observed in 38% (38/100) of the paired adjacent non-neoplastic tissues. TRAF2 expression was greatly higher in GC tissues when compared with that in adjacent counterparts (*p *< 0.05, Fig. 6c).

The relationship between CHIP expression and the clinicopathological characteristics of GC patients was summarized in Table 1. The expression level of CHIP was not correlated with age, gender, tumor diameter, site of tumor, histological types, and tumor differentiation of GC patients. Among T1–T2 and T3–T4 stage samples, the number of low CHIP expression were 4 and 62, indicating that the CHIP expression was negatively correlated with tumor progression (*p *< 0.001). Low CHIP expression occurred more frequently in patients in N2–N3 stage samples (45/66) than in N0–N1 (21/66), indicating that the CHIP expression was significantly correlated with lymph node metastasis (*p *= 0.004). Low CHIP expression was more frequently observed in advanced clinical stage than in early stage samples.

**Table 1:** The correlation of CHIP and TRAF2 expression with the clinical characteristics of GC patients

Characteristics	Nr. of patients n = 100	CHIP expression			TRAF2 expression		
		Low n = 66 (66%)	High n = 34 (34%)	*p* value	Low n = 47 (47%)	High n = 53 53%)	*p* value
Age (years)				0.158			0.106
Median	65 (32–81)						
≤ 65	51	37 (72.5)	14 (27.5)		28 (54.9)	23 (45. 1)	
> 65	49	29 (59.2)	20 (40.8)		19 (38.8)	30 (61.2)	
Gender				0.225			0.102
Male	64	45 (70.3)	19 (29.7)		34 (53.1)	30 (46.9)	
Female	36	21 (58.3)	15 (41.7)		13 (36.1)	23 (63.9)	
Tumor diameter (cm)				0.327			0.067
≤ 5	52	32 (61.5)	20 (38.5)		29 (55.8)	23 (44.2)	
> 5	48	34 (70.8)	14 (29.2)		18 (37.5)	30 (62.5)	
Site of tumor				0.341			0.064
Cardia	13	6 (46.2)	7 (53.8)		4 (30.8)	9 (69.2)	
Body	28	21 (75)	7 (25)		16 (57.1)	12 (42.9)	
Antrum	51	34 (66.7)	17 (33.3)		26 (51.0)	25 (49.0)	
Unknown	8	5 (62.5)	3 (37.5)		1 (12.5)	7 (87.5)	
Histological types				0.541			0.004**
Tubular adenocarcinoma	66	41 (62.1)	25 (37.9)		26 (39.4)	40 (60.6)	
Mucinous adenocarcinoma	10	8 (80.0)	2 (20.0)		8 (80.0)	2 (20.0)	
Signet ring cell adenocarcinoma	11	7 (63.6)	4 (36.4)		9 (81.8)	2 (18.2)	
Undifferentiated carcinoma	13	10 (76.9)	3 (23.1)		4 (30.8)	9 (69.2)	
Tumor differentiation				0.133			0.017*
Well	15	8 (53.3)	7 (46.7)		10 (66.7)	5 (33.3)	
Moderate	74	49 (66.2)	25 (33.8)		35 (47.3)	39 (52.7)	
Poor	11	9 (81.8)	2 (18.2)		2 (18.2)	9 (81.8)	
T classification				0.000***			0.005**
T1–T2	15	4 (26.7)	11 (73.3)		12 (80.0)	3 (20.0)	
T3–T4	85	62 (72.9)	23 (27.1)		35 (41.2)	50 (58.8)	
N classification				0.004**			0.000***
N0–N1	42	21 (50.0)	21 (50.0)		30 (71.4)	12 (28.6)	
N2–N3	58	45 (77.6)	13 (22.4)		17 (29.3)	41 (70.7)	
M classification				0.250			0.056
M0	91	58 (63.7)	33 (36.3)		46 (50.5)	45 (49.5)	
M1	9	8 (88.9)	1 (11.1)		1 (11.1)	8 (88.9)	
TNM stage				0.001***			0.000***
I–II	42	20 (47.6)	22 (52.4)		32 (76.2)	10 (23.8)	
III–IV	58	46 (79.3)	12 (20.7)		15 (25.9)	43 (74.1)	

* *p* < 0.05, ** *p* < 0.01, *** *p* < 0.001

The relationship between TRAF2 expression and the clinicopathological characteristics of GC patients was also summarized in Table 1. There was no significant association between TRAF2 expression and age, gender, tumor diameter, site of tumor, lymph node metastasis (*p *> 0.05). TRAF2 was expressed at significantly higher levels in tubular adenocarcinoma. High level of TRAF2 expression was more frequently observed in poor differentiation samples. TRAF2 expression was positively associated with advanced T, advanced N, and clinical staging (*p *< 0.05). Additionally, the Spearman correlation analysis revealed a significant negative correlation between the expression of CHIP and TRAF2 in the GC tissues (r = − 0.212, *p *= 0.034, Table 2).

**Table 2:** The correlation between the expression of CHIP and TRAF2 in GC tissues

	TRAF2-high	TRAF2-low	Spearmen	p value
CHIP-high	13	21	− 0.212	0.034
CHIP-low	40	26		

Kaplan–Meier analyses indicated that GC patients with high-CHIP expression were significantly correlated with a better OS than those with low-CHIP expression (*p *= 0.01, Fig. 6d). Meanwhile, GC patients with high-TRAF2 expression were significantly correlated with a poor OS than those with low-TRAF2 expression (*p *= 0.01, Fig. 6e).

As shown in Table 3, OS was not significantly correlated with age, gender, site of tumor or histological types according to univariate Cox regression analysis. Importantly, short OS was correlated with large tumor diameter (HR 1.845, *p *= 0.012), poor differentiation (HR 1.978, *p *= 0.005), advanced T (HR 2.483, *p *= 0.034), advanced N (HR 2.039, *p *= 0.005), advanced M (HR 4.766, *p *< 0.001), advanced TNM stage (HR 2.205, *p *< 0.001), low CHIP expression (HR 0.497, *p *= 0.013), and high TRAF2 expression (HR 2.970, *p *< 0.001).

**Table 3:** Univariate and multivariate analyses of OS in 100 patients with GC

Characteristics	Univariate analysis		Multivariate analysis	
	HR (95% CI)	*p* value	HR (95% CI)	*p* value
Age (≤ 65 vs. > 65)	1.005 (0.980–1.031)	0.680	–	–
Gender (male vs. female)	1.202 (0.741–1.950)	0.456	–	–
Tumor diameter (≤ 5 cm vs. > 5 cm)	1.845 (1.146–2.970)	0.012*	1.814 (1.046–3.146)	0.034*
Site of tumor (cardia vs. body vs. antrum vs. unknown)	1.060 (0.832–1.351)	0.638	–	–
Histological types	0.980 (0.788–1.218)	0.856	–	–
Tumor differentiation (poorly vs. moderately, well)	1.978 (1.235–3.167)	0.005**	–	–
T stage (T3 T4 vs. T1 T2)	2.483 (1.072–5.750)	0.034*	–	–
N stage (N3 N4 vs. N0 N1)	2.039 (1.238–3.357)	0.005**	–	–
M stage (M1 vs. M0)	4.766 (2.291–9.916)	0.000***	–	–
TNM stage (III IV vs. I II)	2.205 (1.540–3.159)	0.000***	–	–
CHIP expression (high vs. low)	0.497 (0.287–0.862)	0.013*	–	–
TRAF2 expression (high vs. low)	2.970 (1.801–4.898)	0.000***	2.071 (1.087–3.946)	0.022*

* *p* < 0.05, ** *p* < 0.01, *** *p* < 0.001

TRAF2 expression and tumor diameter was statistically significant correlated with OS, with HR 2.071 for TRAF2 (95% CI 1.087–3.94, *p *= 0.022) and HR 1.814 for tumor diameter (95% CI 1.046–3.146, *p *= 0.034) according to multivariate Cox regression analysis, indicating the TRAF2 expression together with tumor diameter were independent prognostic factors in GC.

## Discussion

Diverse reports have pointed out that CHIP plays contrast roles in individual cancers. CHIP is involved in carcinogenesis, migration, and invasion in quite a few malignancies, regulating a number of oncogenic proteins including hypoxia-inducible factor-1α (HIF-1α), estrogen receptor α (ERα), and human telomerase reverse transcriptase. CHIP is also implicated in the modulation of tumor suppressors including apoptosis-like p53, apop-tosis-inducing factor (AIF), and interferon regulatory factor 1 (IRF-1) [15]. The inconsistence is because of the diversity of CHIP targeting molecules. Recently, a few reports have identified the role of CHIP in modulation of GC carcinogenesis. The poorly differentiated GC tissues have low CHIP expression than the well- and moderate-differentiated samples. The diminished CHIP expression is connected to the clinically aggressive features of GC [14]. CHIP suppresses the potentials of GC cell adhesion and invasion, which are major procedures of tumor metastasis. CHIP expression is negatively correlated with GC progression, such as TNM stage. CHIP is therefore served as an independent prognostic factor for GC patients [6]. According to the previous reports, CHIP is considered as a tumor suppressor in GC.

In the current study, we found that the CHIP-silencing in the AGS gastric cancer cells significantly enhanced cell growth, owing to elevated cell proliferative activity, less apoptosis, and promoted G_1_/S phase transition. The CHIP-silencing in the AGS cells activated the ERK signaling rather than the AKT signaling, contributing to the enhanced cell proliferation capability. The CHIP-silencing inhibited the AGS cell apoptosis due to increased expression of Bcl-2, which is in line the previous report that CHIP-knockdown can regulate the Bcl-2 expression level in breast cancer [16]. The CHIP-silencing notably promoted the G_1_/S phase transition and entry into the S-phase, which was due to the dysregulated Cyclin D1, Cyclin D3, and p53. Collectively, the CHIP-silencing enhanced AGS cell growth, owing to induced proliferative activity, deduced apoptosis, and induced G1/S phase transition. In addition, the CHIP-silencing clearly enhanced the abilities of migration and invasion of the AGS cells, which was associated with the increased expression of Integrin β-1. These findings were certainly in line with the previous findings including our data using the CHIP-overexpressing cell line, which further confirmed that CHIP functions as a tumor suppressor in GC [13].

It has been reported that CHIP could bind to NF-κB subunit RelA and trigger its ubiquitination and proteasomal mediated degradation, which further terminate the canonical NF-κB activity and suppress IL-8-induced angiogenesis in GC cells [6]. NF-κB has been identified as an important transcription factor binding to the κ light chain enhancer in B cells in 1986. The NF-κB family members comprise RelA, RelB, c-Rel, NF-κB1 (p50 and its precursor p105), and NF-κB2 (p52 and its precursor p100). NF-κB can be activated via the canonical signaling pathway that is characterized by the activation of RelA-p50 heterodimers, and the non-canonical signaling pathway that is characterized by the activation of RelB-p52 heterodimers [17]. Activation of the canonical NF-κB cascade requires an adaptor molecule, TRAF2, which is generally considered as a K63-specific E3 ubiquitin ligase. TRAF2 is rearranged and amplified in 15% of human epithelial cancers including GC, and contribute to the constitutively activated NF-κB cascade [18]. The most common genetic alterations of TRAF2 are deep deletion, gene amplification, and mutation. Truncation and fusion of TRAF2 are relatively rare but also detected in human cancers [19]. Genetic alterations of TRAF2 are detected in 1–2% of human hepatocellular carcinoma (HCC). In HCC, low TRAF2 expression and its interacting partner receptor-interacting protein kinase 1 (RIPK1) is correlated with a poor prognosis, suggesting that TRAF2 collaborates RIPK1 with to inhibit hepatocarcinogenesis [20, 21]. TRAF2 also acts as a tumor suppressor in B lymphocytes primarily by inhibiting the NF-κB2 pathway through the cIAP1/2-TRAF2-TRAF3-NIK axis [22]. Interestingly, accumulating evidence point out that TRAF2 is a bona fide oncogene, which is essential for the proliferation and transformation of several types of epithelial cancer cell lines. TRAF2 is also an important biological suppressor of necroptosis in vitro and in vivo [23]. TRAF2 expression is elevated in prostate, pancreatic, lung, and colon cancer than in normal tissues. Suppression of TRAF2 expression inhibits NF-κB activation, which leads to decreased cell proliferation, anchorage-independent growth, and tumorigenesis [18]. Increased TRAF2 expression is recognized as a prognostic factor in several cancers. The exact role of TRAF2 in a specific cancer type is likely dependent on the genetic alteration context, malignant stage of cancer, the nature of the environmental cue, and treatment regimen.

In bladder urothelial carcinoma (BC), receptor-interacting protein kinase 4 (RIPK4) promotes TRAF2 and subsequent NF-κB signaling pathway, ultimately, promotes BC cell aggressiveness [24]. GC patients with TRAF2 hypomethylation live much shorter than those with TRAF2 hypermethylation. Cox regression analysis reveals that TRAF2 hypomethylation, lymph node metastasis, distant metastasis, as well as differentiation are vital prognostic factors in GC. Therefore, TRAF2 expression is increased in GC patients by DNA hypomethylation and this methylation could be an independent diagnostic and prognostic indicator in GC [25]. It has been shown that TRAF2 expression is markedly increased in GC tissues, which is associated with tumor invasion and metastasis. High TRAF2 expression can enhance NF-κB activation and subsequent IL-8 expression in GC cells. TRAF2 is an independent poor prognosticator for GC patients [26]. In MDA-MB-231 breast cancer cells, TRAF2 has been found to be a substrate for CHIP and that, consequently, CHIP regulates cell invasion via ubiquitination and degradation of TRAF2 and further inhibition of the NF-κB activation [27]. Although the expression of TRAF2 was not affected by the CHIP-silencing in the AGS cells according to current study, TRAF2 expression at protein level is indeed reduced in the CHIP-overexpressing AGS cells [13]. Therefore, TRAF2 expression was downregulated at the translational level but not the transcription level in the presence of CHIP-overexpession in the AGS cells. In the current study, we established a stable TRAF2-silencing AGS cells line. The expression of all subunits of the NF-κB family members was clearly decreased in the absence of TRAF2 expression. Meanwhile, the NF-κB DNA-binding activities in the nucleus were markedly deduced. The TRAF2-silencing inhibited AGS cell growth, proliferation, migration and invasion, indicating that TRAF2 played an oncogenic role in GC cells. These results were consistent with the results obtained from the CHIP-overexpressing AGS cells and were contrast to the results obtained from the CHIP-silencing AGS cells. Therefore, it is possible that TRAF2 was a substrate of CHIP and CHIP regulated the TRAF2/NF-κB axis, which modulated diverse cellular behaviors of the AGS gastric cancer cells.

In the present study, the expression of CHIP was significantly reduced in GC tissues compared with adjacent non-neoplastic tissue, while the expression of TRAF2 was elevated. The expression level of CHIP was negatively associated with depth of tumor invasion, lymph nodes invasion, and TNM stage. The expression level of TRAF2 was positively associated with degree of differentiation, depth of tumor invasion, lymph nodes invasion, and TNM stage. According to the Spearman correlation analysis, a significant negative correlation between the expression of CHIP and TRAF2 was observed in the GC tissues. These results suggested that low CHIP expression together with high TRAF2 expression played important roles in GC tumor progression and metastasis, in line with the in vitro findings. In addition, lower CHIP expression or higher TRAF2 expression were significantly correlated with shorter OS in GC patients. Importantly, according to multivariate Cox regression analysis, TRAF2 expression together with tumor diameter was independent prognostic factors in GC patients. The potential mechanism by which CHIP negatively regulates the expression of TRAF2 remains unclear. Ubiquitination and proteasome-mediated degradation of TRAF2 by CHIP might be one of the explanations.

## Conclusions

TRAF2 influenced diverse aspects of cellular behavior of GC cells, including cell growth, migration, and invasion, which was in contrast to the functions of CHIP in GC cells. The expression of CHIP and TRAF2 was negatively correlated in the GC tissue. Lower CHIP or higher TRAF2 was significantly linked to shorter OS in GC patients. TRAF2 is an independent prognostic factor in GC patients. In conclusion, our study indicated that TRAF2 is a potential target of CHIP in GC cells. CHIP regulated the TRAF2/NF-κB axis, which modulated diverse cellular behaviors of the AGS gastric cancer cells.

## Additional file

**Additional file 1: Fig. S1.** Establishing a CHIP-silencing cell line. **a** *CHIP* mRNA expression between the two established cell lines. *β-actin* normalized gene expression, measured in triplicates was displayed. Significant differences were indicated (Student’s *t*-test, ***p<0.001). **b** Protein levels of CHIP expression in the two established cell lines were determined by Western blotting analysis. Actin was used as an internal control. **Fig. S2.** The expression levels of proteins in the AKT signaling pathway were analyzed by Western blotting analysis. Actin was used as an internal control. **Fig. S3.** Establishing a TRAF2-silencing cell line. **a** *TRAF2* mRNA expression between the two established cell lines. *β-actin* normalized gene expression, measured in triplicates was displayed. Significant differences were indicated (Student’s *t*-test, ***p<0.001). **b** Protein levels of TRAF2 expression in the two established cell lines were determined by Western blotting analysis. Actin was used as an internal control.

## Data Availability

The datasets supporting the conclusions of this article are included within the article.

## Abbreviations

GC: gastric cancer
HER-2: human epidermal growth factor receptor-2
VEGFR2: anti-vascular endothelial growth factor receptor 2
OS: overall survival
CHIP: carboxyl terminus of Hsc70-interacting protein
TPR: tetratrico peptide repeat
HSP70: chaperones heat shock protein 70
HSP90: chaperones heat shock protein 90
EGFR: epidermal growth factor receptor
FBS: fetal bovine serum
shRNA: short hairpin RNA
PBS: phosphate-buffered saline
Ab: antibody
DAB: 3,3-diaminobenzine
CCK-8: cell counting kit-8
TUNEL: TdT-mediated dUTP nick-end labeling
PI: propidium iodide
CFSE: carboxy fluorescein diacetate succinimidylester
qRT-PCR: quantitative real-time PCR
IHC: immunohistochemistry
SD: standard deviation
OD: optical density
AKT: protein kinase B
PTEN: tension homology deleted on chromosome ten
ERK: extracellular regulated protein kinases
Bcl-2: B-cell lymphoma-2
BIM: Bcl-2 interacting mediator of cell death
CDK4: cyclin-dependent kinase 4
CDK6: cyclin-dependent kinase 6
uPA: urokinase plasminogen activator
MMP2: matrix metallopeptidase 2
MMP9: matrix metallopeptidase 9
TRAF2: TNF receptor-associated factor 2
NF-κB: nuclear factor kappa-light-chain-enhancer of activated B cells
NE: nuclear extracts
CE: cytoplasmic extracts
HIF-1α: hypoxia-inducible factor-1α
ERα: estrogen receptor α
AIF: apoptosis-inducing factor
IRF-1: interferon regulatory factor 1
HCC: hepatocellular carcinoma
RIPK1: receptor-interacting protein kinase 1
BC: bladder urothelial carcinoma
RIPK4: receptor-interacting protein kinase 4
