# Supplementary material for: The ubiquitin ligase CHIP modulates cellular behaviors of gastric cancer cells by regulating TRAF2
Source: Cancer Cell Int. 2019 May 16;19:132. doi: 10.1186/s12935-019-0832-z (PMC6524225; doi:10.1186/s12935-019-0832-z)

**Fig. S1** Establishing a CHIP-silencing cell line. **a** *CHIP* mRNA expression between the two established cell lines. *β-actin* normalized gene expression, measured in triplicates was displayed. Significant differences were indicated (Student's *t*-test, ****p*<0.001). **b** Protein levels of CHIP expression in the two established cell lines were determined by Western blotting analysis. Actin was used as an internal control.

A

B


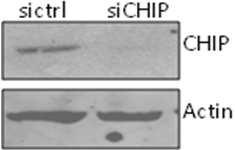


**Fig. S2** The expression levels of proteins in the AKT signaling pathway were analyzed by Western blotting analysis. Actin was used as an internal control.


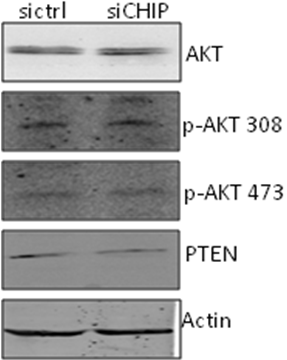


**Fig. S3** Establishing a TRAF2-silencing cell line. **a** *TRAF2* mRNA expression between the two established cell lines. *β-actin* normalized gene expression, measured in triplicates was displayed. Significant differences were indicated (Student's *t*-test, ****p*<0.001). **b** Protein levels of TRAF2 expression in the two established cell lines were determined by Western blotting analysis. Actin was used as an internal control.

A

B


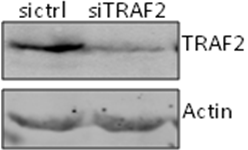

Supplement: Supplementary file 1 — Additional file 1: Fig. S1. Establishing a CHIP-silencing cell line. a CHIP mRNA expression between the two established cell lines. β-actin normalized gene expression, measured in triplicates was displayed. Significant differences were indicated (Student’s t-test, ***p<0.001). b Protein levels of CHIP expression in the two established cell lines were determined by Western blotting analysis. Actin was used as an internal control. Fig. S2. The expression levels of proteins in the AKT signaling pathway were analyzed by Western blotting analysis. Actin was used as an internal control. Fig. S3. Establishing a TRAF2-silencing cell line. a TRAF2 mRNA expression between the two established cell lines. β-actin normalized gene expression, measured in triplicates was displayed. Significant differences were indicated (Student’s t-test, ***p<0.001). b Protein levels of TRAF2 expression in the two established cell lines were determined by Western blotting analysis. Actin was used as an internal control. [file 12935_2019_832_MOESM1_ESM.doc]
